# Characterizing the gut microbiome changes with aging in a novel Alzheimer’s disease rat model

**DOI:** 10.18632/aging.204484

**Published:** 2023-01-13

**Authors:** Akash Nagarajan, Hemant Srivastava, Casey D. Morrow, Liou Y. Sun

**Affiliations:** 1 Department of Biology, University of Alabama at Birmingham, Birmingham, AL 35254, USA; 2 Department of Cell, Developmental and Integrative Biology, University of Alabama at Birmingham, Birmingham, AL 35294, USA

**Keywords:** Alzheimer’s disease, gut microbiome, gut-brain axis, aging, F344 rats

## Abstract

Alzheimer’s disease (AD) is one of the most devastating diseases currently in the world with no effective treatments. There is increasing evidence that the gut microbiome plays a role in AD. Here we set out to study the age-related changes in the microbiome of the Tgf344-AD rats. We performed 16S ribosomal RNA sequencing on the fecal samples of male rats at 14 and 20 months of age. We found the Tgf344-AD rats to have decreased microbial diversity compared to controls at 14 months of age and this was found to be opposite at 20 months of age. Interestingly, we found a distinctive shift in the microbial community structure of the rats with aging along with changes in the microbiota composition. Some of the observed changes in the Tgf344AD rats were in the genera *Bifidobacterium, Ruminococcus, Parasutterella, Lachnoclostridium* and *Butyricicoccus*. Other age-related changes occuring in both the Tgf344-AD and WT control rats were decreases in *Enterohaldus, Escherichia Shigella, Rothia* and increase in *Turicibacter* and *Clostrium_senso_stricto*. Our study has shown that gut microbiota changes occurs in this Alzheimer’s disease rat model.

## INTRODUCTION

The gut microbiome has been found to be altered in patients with neurodegenerative diseases such as Alzheimer’s disease [1], multiple sclerosis [2], Parkinson’s disease [3] and psychiatric disorders such as depression, anxiety and autism spectrum disorder [4]. The gut brain axis is a well-coordinated network between the intestinal microbiota, enteric nervous system (ENS) and the central nervous system (CNS) and they communicate with each other through neural, endocrine and immune mediators [5]. Understanding more about the gut microbiota changes associated with neurodegeneration and aging may help us in elucidating the complex crosstalk between the gut and the brain. Alzheimer’s disease (AD) is the most common form of neurodegenerative disease which affects primarily individuals of old age. Aging is the greatest risk factor for Alzheimer’s disease and chances of developing dementia or AD increases exponentially in the later stages of life (>65years) [6]. Both Alzheimer’s disease and biological aging have shown to cause gut microbiome alterations [1, 7].

There is evidence from rodent models of disease and human patients that AD may be linked with gut microbiome changes. A study in an AD mice model has also shown that gut microbiota alterations are observed in those mice [8]. Two recent fecal matter transplant (FMT) studies in AD mice models have shown that it can improve memory and reduce beta amyloid pathology in the brain [9, 10]. Recent clinical studies in human AD patients from 2 different geographical locations have also shown that gut microbiota changes are associated with Alzheimer’s disease [11]. There was also a recent report of dramatic reduction in Alzheimer’s disease symptoms in a patient after fecal matter transplantation to treat *C. difficile* infection [12]. These studies posit a view that gut microbiome modulation could be a potential therapy for treating AD.

Gut microbiome composition changes have been found to occur with aging in many different species of animals including humans. Whether they play a causal role in the aging process is uncertain. The gut microbiota has been shown to modulate the lifespan in short-lived vertebrates and a progeroid mouse model. This was demonstrated by fecal matter transplantation studies of young microbiome in killifish [13] and progeroid mice [14] which resulted in an extension of lifespan. These studies show that the microbiome may play an important role in the aging process.

Here we have characterized the gut microbiota changes occurring due to the Alzheimer’s pathology along with aging associated changes in the Tgf344-AD rat model [15]. The Tgf344-AD rat model was developed in 2013 by Cohen et al. and since then many different studies have been published studying various aspects of AD using this rat model. This rat model demonstrates some unique characteristics compared to other rat models such as manifestation of neurofibrillary tangles in the absence of other human tau mutations and also consistent and extensive neuronal loss. These rats show progressive neurodegeneration and amyloidosis in the brain precedes other disease pathology events such as tauopathy, gliosis and neuronal death. This rat model shows pathology and behavioral changes by 14 months of age and this is the first time point we have chosen to study the microbiome changes. We used male Tgf344-AD rats and wildtype control rats for our study and fecal samples were collected at 14 and 20 months of age for analyses.

## METHODS

### Animals

Breeder rat pairs of TgF344-AD rats (wild-type females and transgenic males) were obtained from Dr. Terrence Town of USC and these were bred together create the initial litter and the subsequent experimental colony. All rats were maintained in a normal 12-h light/dark cycle with ad libitum access to food and water. All animals were housed at Research Support Building (RSB) University of Alabama, Birmingham. All experiments and procedures were conducted in compliance with protocols approved by the Institutional Animal Care and Use Committee (IACUC). All the rats were weaned at 3 weeks of age and tail clips were taken for genotyping. Genotyping by PCR on the transgenes was performed as previously described. Transgenic and wild-type rats were cohoused together.

### Fecal sample collection

Fecal pellets were collected from the Tgf344-AD and WT control rats at ages: 14 months and 20 months of age (WT14M, AD14M, WT20M, AD20M). Only male rat samples were chosen for this study. The fecal pellets were collected at the same time of the day for consistency. Rats were moved to individual autoclaved cages and housed separately at the time of sample collection. The cages which were sterilized before each rat was placed in it. 2–3 fecal pellets were collected per rat. The fecal pellets were placed into 1.5 mL tubes and immediately stored in ice. All the collected fecal pellets were transported to the −80°C freezer within 2 hours of collection. The fecal samples were transferred to the UAB Microbiome Resource before DNA extraction, where they were stored at a −20°C freezer.

### DNA extraction and 16s rRNA sequencing

DNA extraction process was performed on the chosen samples using the Fecal DNA Isolation Kit from Zymo Research according to the manufacturer’s instructions. The isolated DNA was then used for PCR or stored in Tris-EDTA buffer for later use. The DNA was quantified in a Spectrophotometer before PCR. PCR amplification of the V4 region of the 16S rRNA gene was carried out using unique barcoded primers to create an amplicon library.

The individual PCR products were run on an Agarose gel, bands visualized by UV illumination and they were in turn excised and purified by QIAquick Gel Extraction Kit (Qiagen, Germantown, MD, USA). The PCR products were sequenced using the Illumina MiSeq platform by 250 bp paired-end sequencing.

### Bioinformatic and statistical analysis

Demultiplexed data files was obtained from the UAB Microbiome Resource after the sequencing process. In the first step of the analysis, the FASTQC files were imported into the Quantitative insights for microbial ecology 2 (QIIME2) environment as qza files using the “import” function and the manifest method. DADA2 denoising algorithm was used to cluster the sequence at 99% similarity. DADA2 workflow implements the following steps: filtering of the reads, dereplication, chimera removal and merging of paired-end reads. The final output from DADA2 is an ASV table and the representative sequences file containing the ASV ID matching with an amplicon sequence read. A Phylogenetic tree was constructed using the q2-phylogeny plugin and the align-to-tree-mafft-fasttree pipeline. Alpha and Beta diversity analysis were performed in QIIME2 and rarefaction was done to ensure the same number of random sequencing reads were used for all samples. Taxonomic classification of the amplicon reads was performed based on the SILVA database using a naïve Bayes classifier. Faith’s phylogenetic diversity and Observed ASV’s were the two alpha diversity measures calculated. The Kruskal Wallis test was used to compare the alpha diversity measures.

Unweighted Unifrac distance was calculated to assess Beta diversity. Unweighted Unifrac distance matrix were used to create the Principal Coordinate Analysis (PCoA) plot in QIIME2 and in R using phyloseq. Statistical analysis was performed using the Unweighted Unifrac distances between groups using Permutational multivariate analysis of variance (PERMANOVA) testing. Linear discriminant analysis by effect size (LEfSe) was used to determine the differentially abundant taxa between the different groups. LEfSe uses a non-parametric factorial Kruskal Wallis test to detect features which are differentially represented between groups. LEfSe was performed using GALAXY [16] and the results were plotted as a Cladogram and as LDA histograms. As with other analyses, both genotypic and age-related comparisons were made. Phyloseq [17], vegan packages [18] were used in R for various microbiome analyses.

## RESULTS

To investigate the gut microbiota changes in the Tgf344 AD rat model and to study the age-related microbiome changes, we collected and sequenced fecal samples from rats at 2 different ages (14 months and 20 months). Firstly, we focused on (i). The genotypic differences at 14 months and 20 months of age and (ii). The age-related changes for both the WT control rats and the Tgf344-AD rats.

### Richness and diversity of experimental groups

**Figure 1:**
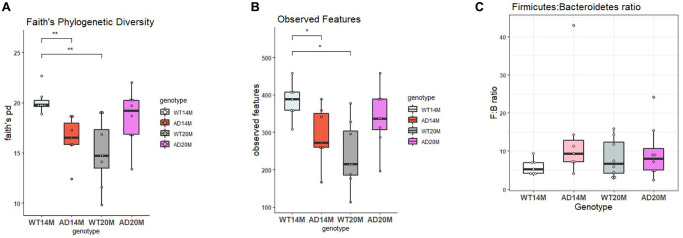
**Richness and diversity of experimental groups.** (**A**) Faith’s Phylogenetic diversity and (**B**) Observed Features for Tgf344-AD and WT control rats at 14 and 20 months of age. (**C**) *Firmicutes:Bacteroidetes* ratio for Tgf344-AD and WT control rats at 14 and 20 months of age. Statistical analysis performed using the Kruskal Wallis test; ^*^p < 0.05, ^**^p < 0.01.

To assess the richness and diversity of the samples, alpha diversity measures was examined. Faith’s phylogenetic diversity and Observed ASV’s were the 2 metrices calculated and comparisons were done to assess both the genotypic changes and age-related changes. The 14-month-old Tgf-344AD rats showed a decrease in both metrics (Figure 1A, 1B), faiths PD and observed Features (Kruskal Wallis test; *p* = 0.0054 and 0.05; Tables 1 and 2), and at the 20-month time point, this was found to be reversed. The WT control rats showed a steep decrease in both alpha diversity metrices at 20 months of age. There was increased diversity in the Tgf344-AD rats when compared to WT control rats (*p* = 0.09 and 0.07; Tables 1 and 2) at 20 months of age but it was not statistically significant. The age-related decrease in both diversity and number of observed ASV’s for the WT animals was significant (*p* = 0.005 and 0.033; Tables 1 and 2) while for the AD rats there is a tendency towards an increase, but it was not statistically significant. The Firmicutes: Bacteroidetes (F:B) ratio has been viewed as a biomarker of intestinal homeostasis and frequently observed with obesity. The Tgf344-AD rats did not show any changes in the Firmicutes:Bacteriodetes ratio when compared to WT control rats at both the ages (Figure 1C).

**Table 1:** Faith’s PD statistical analysis.

**Comparison**	**H value**	***p*-value**	***q*-value (adjusted *p*-value)**
AD14M vs. WT14M	9.8	0.001745	0.005341
AD20M vs. WT20M	3.98	0.045	0.091
AD14M vs. AD20M	3.01	0.0826	0.123
WT14M vs. WT20M	9.763	0.00178	0.0053

**Table 2:** Observed ASV’s statistical analysis.

**Comparison**	**H value**	***p*-value**	***q*-value (adjusted *p*-value)**
AD14M vs. WT14M	5.588	0.018	0.054
AD20M vs. WT20M	4.411	0.0357	0.0714
AD14M vs. AD20M	1.21	0.271	0.271
WT14M vs. WT20M	7.714	0.0055	0.0329

	**AD14M**	**WT14M**	**AD20M**	**WT20M**
Faith’s PD	16.44 ± 1.99	20.15 ± 1.12	18.5 ± 2.28	14.97 ± 2.73
Observed ASV’s	292 ± 70	383.57 ± 44.1	338 ± 65.45	238.38 ± 73.23

### Changes in community structure

**Figure 2:**
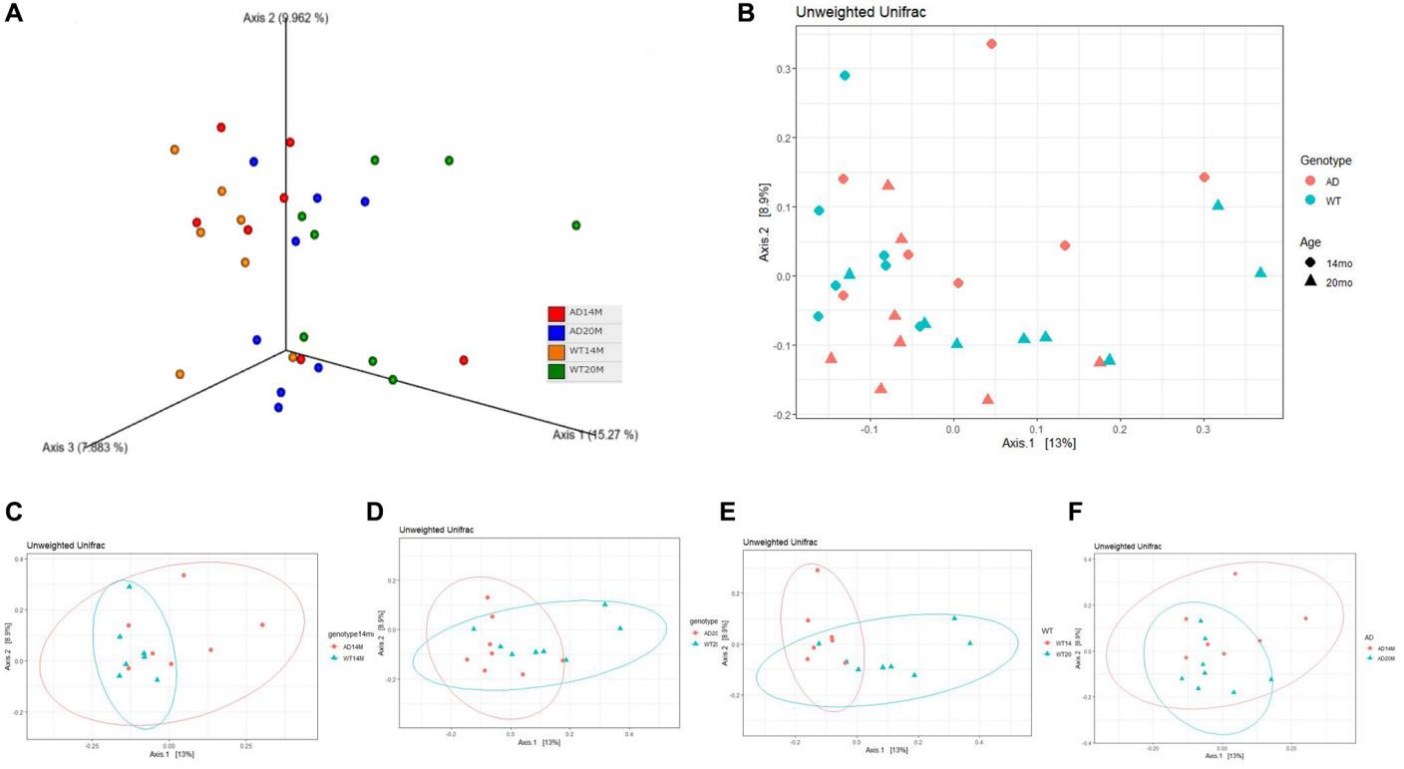
**Changes in community structure.** (**A**) Principal coordinate analysis plot of Unweighted Unifrac distance in a 3 dimensional view and (**B**) 2 dimensional view. PCoA plots stratified to show (**C**, **D**) genotypic changes at 14 and 20 months of age; and (**E**, **F**) aging changes.

To assess the similarity between the overall microbial communities of the groups, Beta diversity indices was calculated using the Unweighted Unifrac distance metric. The three-dimensional visualization of the community structure was done using Principal Coordinates analysis plot (PCoA) carried out using pairwise Unweighted Unifrac distances (Figure 2A). There was a striking change in the microbiota community structure between middle aged and old age animals (Figure 2A–2D). Permutational multivariate analysis of variance (PERMANOVA) was carried out on the distance between groups to analyze if the variation was of statistical significance. The age- related changes was statistically significant for the WT group (*p* = 0.012; Table 3) and showed a trend in the AD group (*p* = 0.078; Table 3) (Figure 2C, 2D). The genotypic changes (Figure 2E, 2F) were not found to be statistically significant.

**Table 3:** Unweighted Unifrac PERMANOVA results.

**Comparison**	**Pseudo-F**	***p*-value**	***q*-value (adjusted *p*-value)**
AD14M vs. WT14M	1.20	0.158	0.158
AD20M vs. WT20M	1.36	0.096	0.1176
AD14M vs. AD20M	1.478	0.026	0.078
WT14M vs. WT20M	2.339	0.002	0.0120

### Taxonomic composition of the gut microbial communities

**Figure 3:**
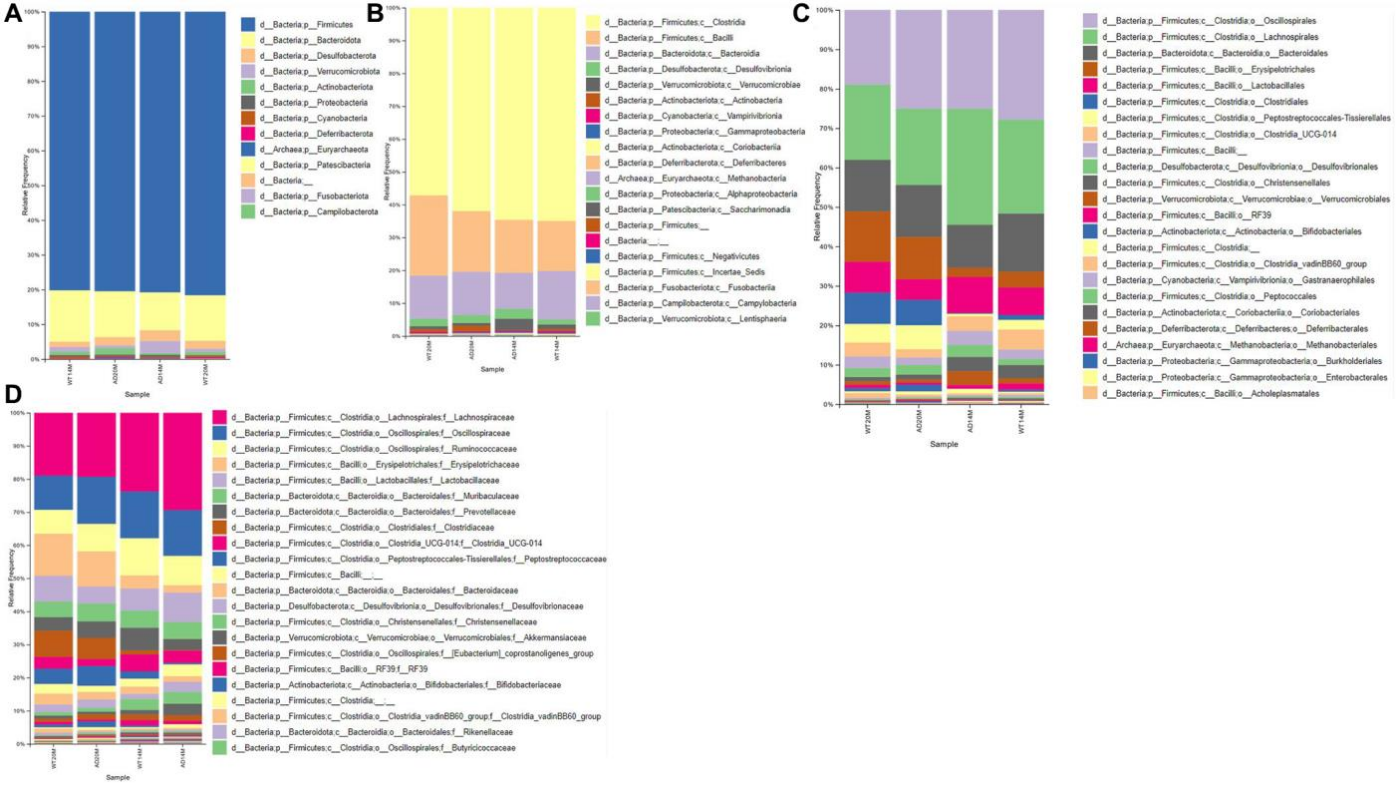
**Taxonomic composition of gut microbial communities.** Composition of gut microbial communities of the Tgf344-AD and WT control rats, visualized at different levels of classification: (**A**) Phylum, (**B**) Class, (**C**) Order, (**D**) Family.

The taxonomic composition of the gut microbial communities at different levels of classification namely: Phylum, Class, Order and Family, are visualized as stacked bar plots (Figure 3A–3D).

### Gut microbiome changes due to genotype

**Figure 4:**
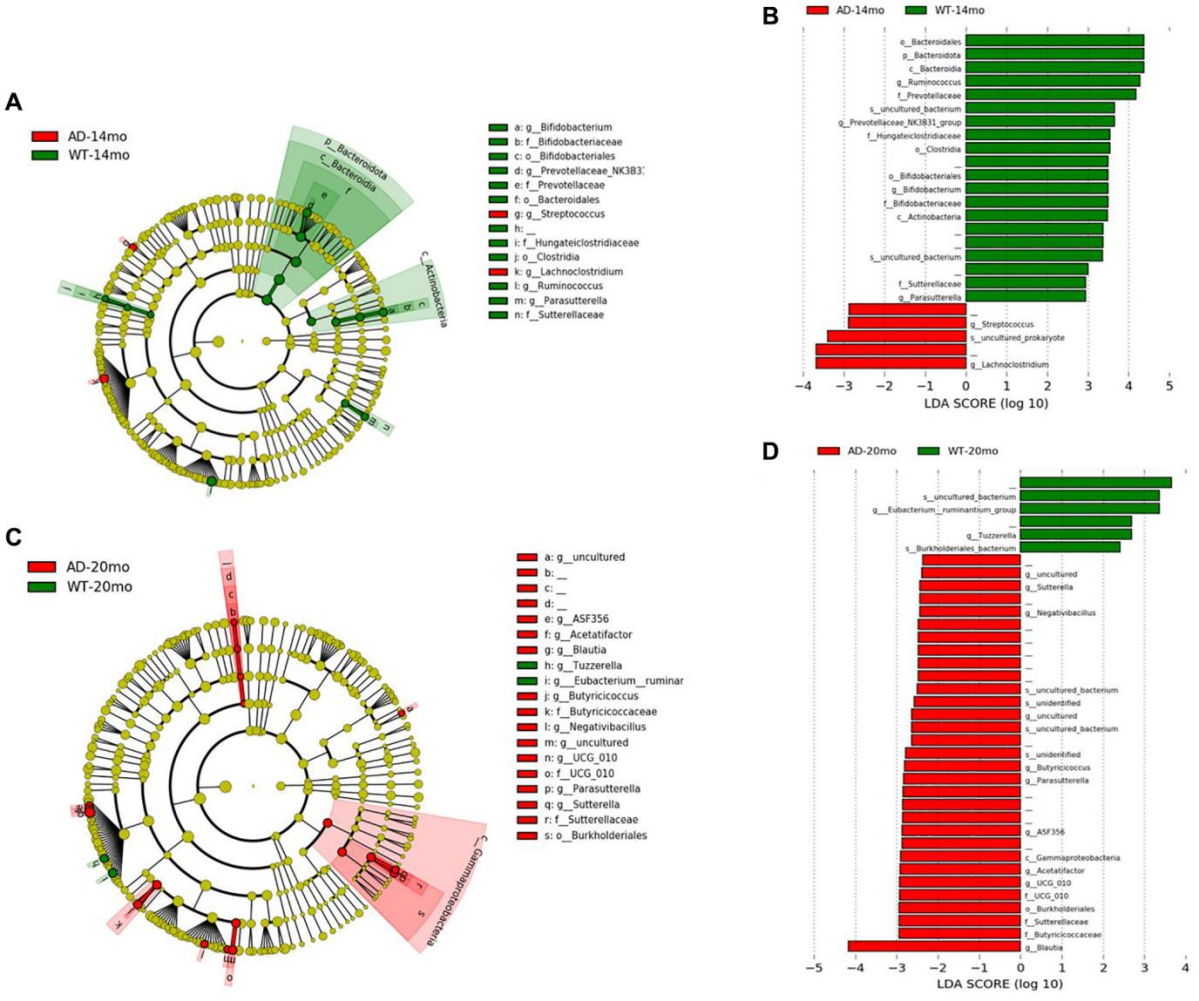
**Gut microbiota changes due to AD pathology.** Microbial taxa that were differentially represented between the Tgf344-AD and WT rats was determined by LEfSe and visualized by Cladogram and LDA histograms at 14 months (**A**, **B**) of age and 20 months (**C**, **D**) of age respectively.

**Figure 5:**
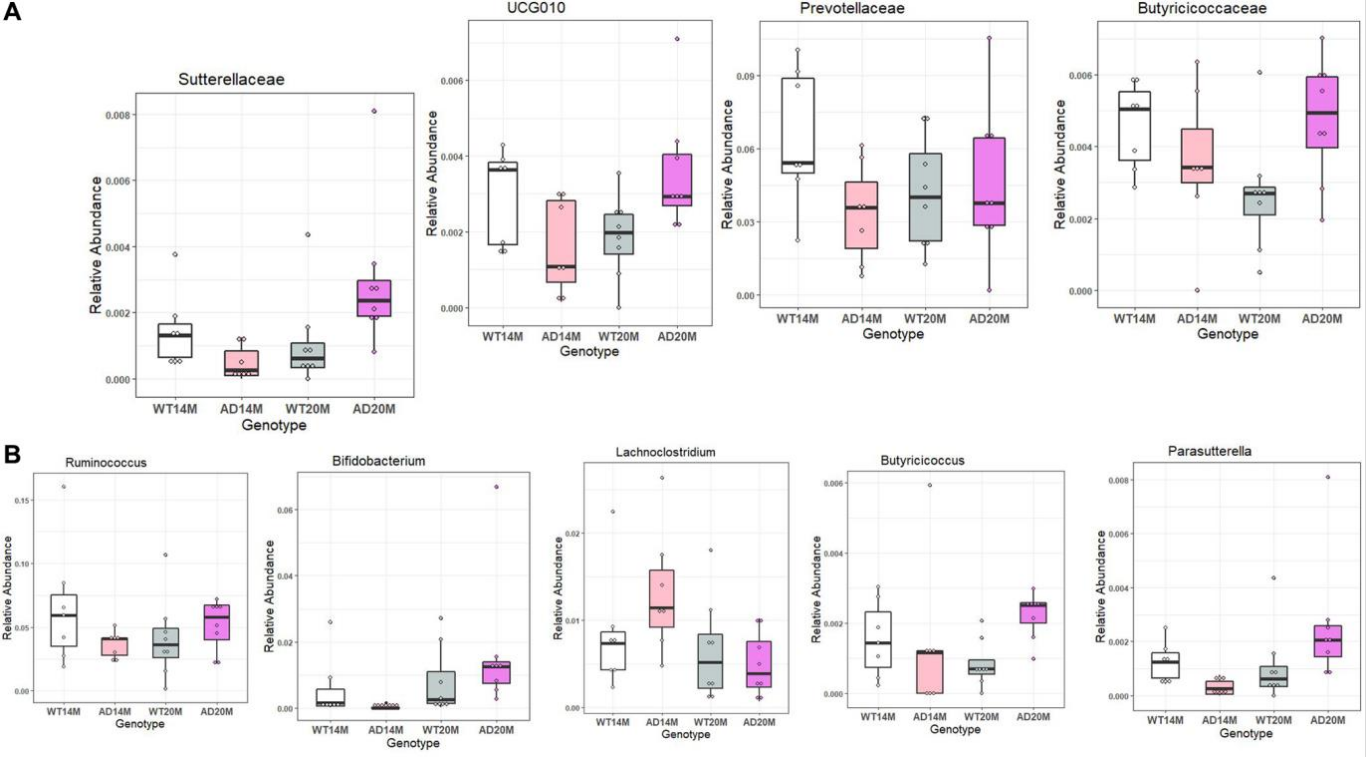
**Differentially represented taxa in the Tgf344-AD rats.** Differentially abundant taxa between the Tgf344-AD and WT rats at the (**A**) Family and (**B**) Genus levels identified using LefSe.

To determine the changes in the gut microbial communities occurring due to Alzheimer’s disease phenotype and aging, LEfSe was performed (Figure 4A, 4C). Bacterial taxa was found to be changed between AD and WT groups at different levels of taxonomic classification starting at the Phylum level. At the Phylum level, *Bacteroidota* was decreased in AD rats at 14 months of age (Figure 4B, 4D). At the Class level, *Bacteroidia* and *Actinobacteria* were decreased in AD rats at 14 months of age. At 20 months of age, *Gammaproteobacteria* was increased in the AD rats group. At Order level, *Bacteroidales, Clostridia* and *Bifidobacteriales* were found to be decreased at 14 months of age while *Burkholderiales* was increased at 20 months of age. Some of the prominent family level genotypic changes were *Prevotellaceae* decreasing in AD group at 14 months and *UCG_010*, *Sutterellaceae, Butyricicoccaceae* increasing at 20 months (Figure 5A). The prominent genotypic changes at the genus level was a decrease in *Ruminococcus, Bifidobacterium, Parasutterella* and increases in *Lachnoclostridium* at 14 months of age (Figure 5B). At 20 months of age, a prominent change was *Butyricicoccus* which was increased.

### Gut microbiome changes occurring with increasing age

**Figure 6:**
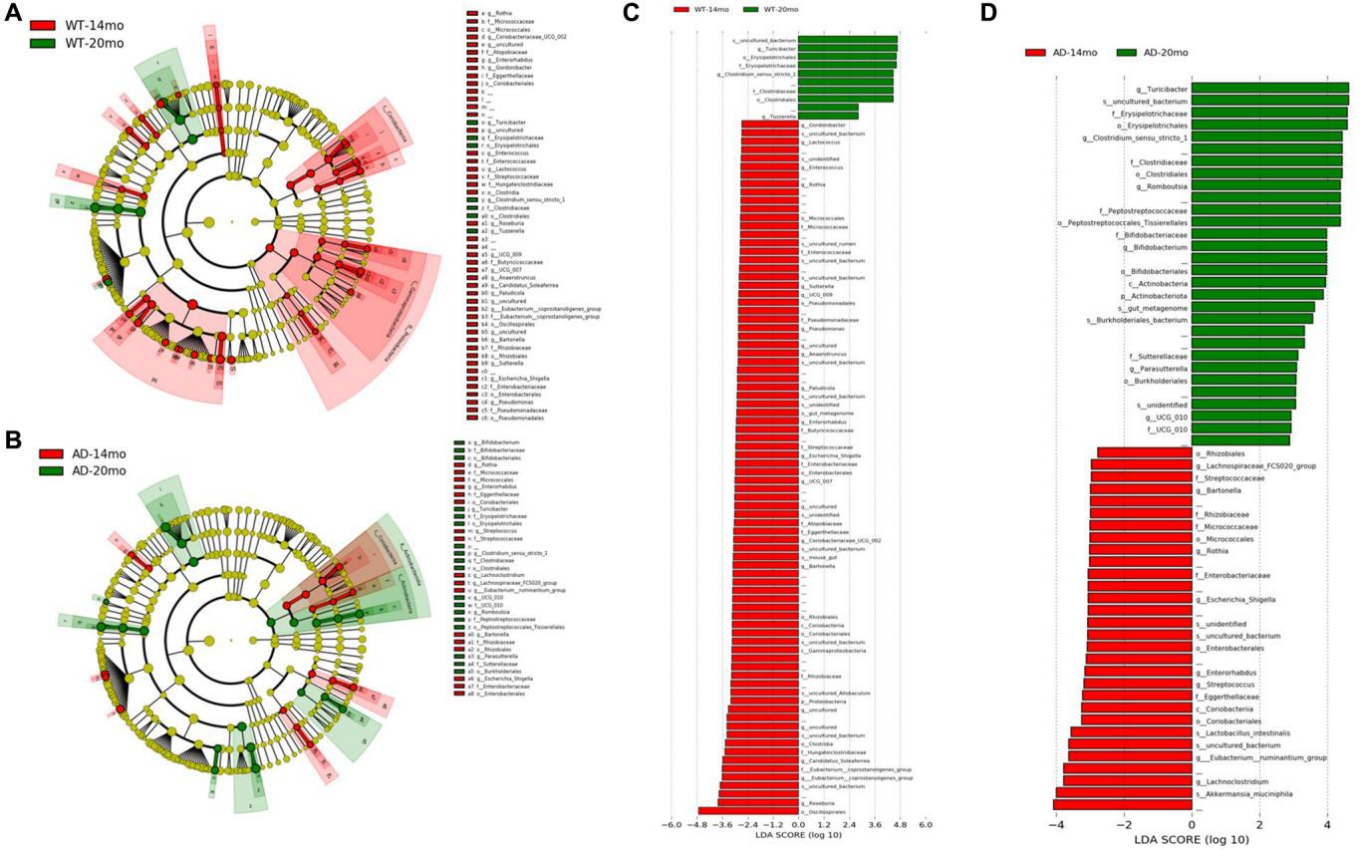
**Gut microbiota changes due to aging.** The effect of aging on the gut microbial communities of the Tgf344-AD rats and WT rats. Microbial taxa that were differentially represented was determined by LEfSe and visualized by Cladogram (**A**, **B**) and LDA histograms (**C**, **D**) at 14 months of age and 20 months of age.

The number of bacterial taxa found to be differentially represented was much higher for age-related changes compared to genotypic changes. For WT age-related change analysis, most of the differentially represented taxa were found to be decreased at 20 months of age (Figure 6A, 6C). This shows us that there was an age-related decrease in the microbiome composition. For the Tgf344-AD rats group, more bacterial taxa were found to be increased at 20 months of age than those that were found to be decreasing, which is in stark contrast to the WT rats group (Figure 6B, 6D). Class *Coriobacteria* was found to be decreased in both the groups with age. At the Order level, *Micrococcales, Coriobacteriales, Rhizobales, Enterobacterales* were found to be reduced in both WT and AD rats (Figure 6B, 6D), while *Erysipelotrichales* and *Clostridiales* were increased with age in both the rat groups.

**Figure 7:**
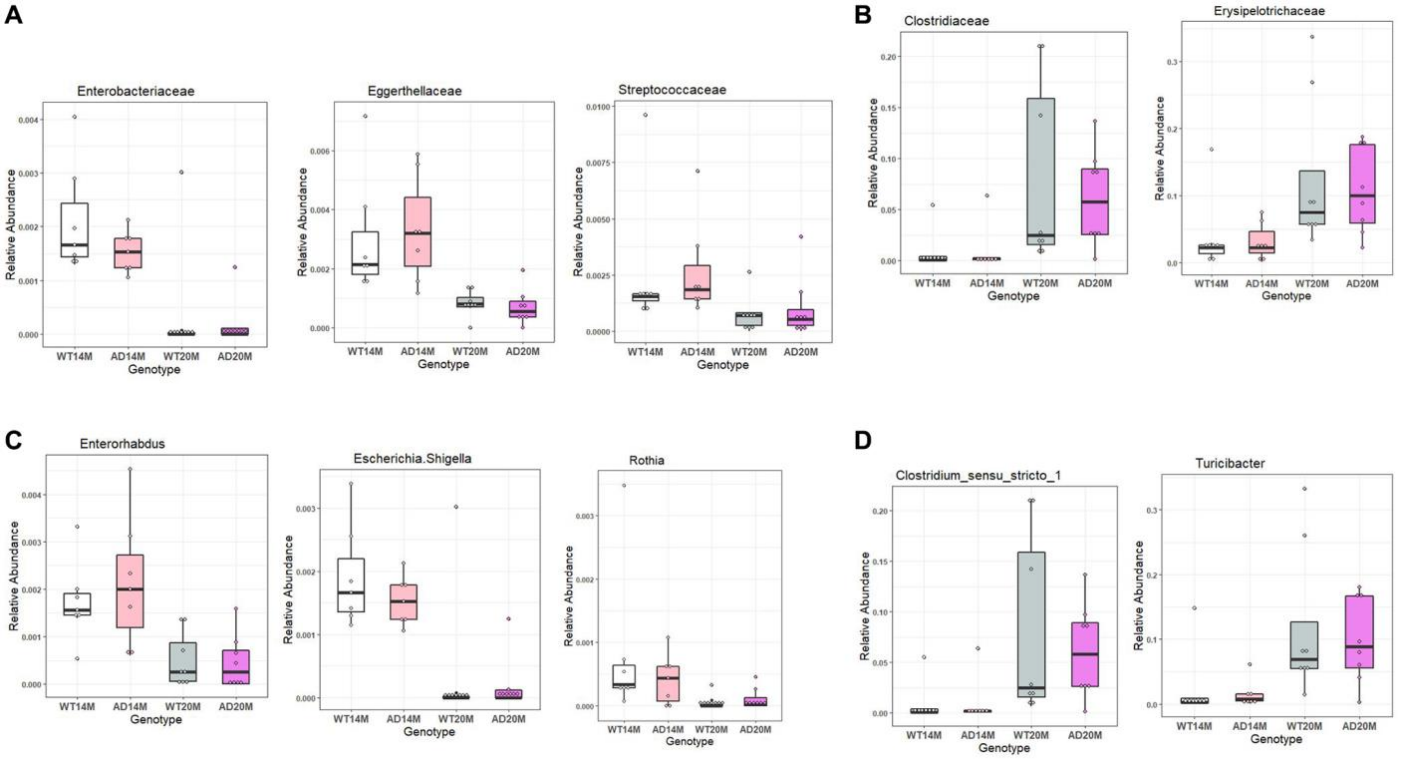
**Conserved aging associated changes between both Tgf344-AD and WT control rats.** Conserved aging-associated changes between the Tgf344-AD and WT rats. (**A**, **B**) Families increasing with age and decreasing with age, (**C**, **D**) Genera increasing with age and decreasing with age identified using LefSe.

Some of the prominent changes at the Family level were *Enterobacteriaceae, Eggerthellaceae and Streptococcaceae* decreasing in both the WT and AD rat groups with age while *Erysipelotrichacae* and *Clostridiaceae* were increased in both the rat groups with age (Figure 7A, 7B).

The prominent changes at genus level were *Rothia, Enterohabdus* and *Escherichia shigella* decreasing in both the AD rats and WT control rats while *Turicibacter* and *Clostrium_sensu_stricto* were found to be increased in them (Figure 7C, 7D).

## DISCUSSION

The gut microbiota has been associated in many diseases including neurodegenerative diseases. The gut brain axis is hypothesized to have a major role in the disease progression and possibly may even play an etiological role. Aging also influences the microbiome diversity, richness, and composition dramatically [19]. In this study, we have demonstrated, altered gut microbiome changes in a novel Alzheimer’s disease rat model, and the dramatic changes and shifts in microbiome composition and structure due to aging in the Fischer 344 rat model. One notable feature we are looking at, are the changes in the later stage of the animal’s life, as Alzheimer’s disease is an aging-associated disease and is seen only in aged people during the last stages of life. We have characterized the gut microbiota changes at a stage in the animal’s life (late adult to old age) similar to when it affects humans.

There is strong evidence from human clinical studies showing that there are gut microbiome alterations seen in a plethora of diseases [20]. Studies done in germ-free mice and antibiotic-treated mice have consolidated this view that the microbiome plays an important role in these diseases and microbiome composition may be required for healthy aging. Fecal matter transplantation studies have shown that many beneficial characteristics of the donor phenotype such as exercise [21], caloric restriction [22–24] can be recapitulated in the recipient thereby showcasing the important role the microbiome plays in maintaining homeostasis. The opposite is also true where transplant from donor with natural or diet-induced obesity [24] (in the case of mice) or various diseases can also recapitulate some of that negative phenotype. There is also evidence that gut microbiota might enhance the AD pathology as seen from a microbiome depletion study in the APP/PS1 mice model done using antibiotics [25]. Diversity was also found to have a negative correlation with frailty [26]. Similar to human Alzheimer’s patients [1], alpha diversity was found to be decreased in our Tgf344-AD rats at 14 months of age but not 20 months where aging might have obscured the changes. As expected with aging, the alpha diversity was again found to decrease with age in our rats. The dramatic change in the community structure due to aging in our rats was also similar to other rodent microbiome aging studies. Beta diversity changes due to genotype were less prominent when compared with aging which might show that the changes due to AD pathology might be subtle compared to system-wide effects of aging.

Diabetes, obesity are risk factors for Alzheimer’s disease, and insulin resistance and disrupted glucose metabolism have been hypothesized to be causal factors in Alzheimer’s disease [27]. *Firmicutes:Bacteriodetes* ratio is a commonly studied variable in microbiome studies and it has been commonly associated with obesity [28]. Changes in the *Firmicutes:Bacteriodetes* ratio has also been observed with aging [29]. We found no change in F:B ratio between groups in our animals including with age where gut dysbiosis has been hypothesized to occur with aging. There have been 2 previous microbiome studies done in human Alzheimer’s disease patients so far [1, 11] and they have shown the diversity to be decreased and alterations in community structure like our Tgf344-AD rats. However there has been differences in the results observed with respect to differentially expressed taxa, the phyla *Bacteroidetes* increased and *Actinobacteria* decreased in one study while the opposite was observed in the other study. This discrepancy may have been due to many other factors such as geographical location, diet, ethnicity etc., Further studies investigating whether SCFA (short chain fatty acids) levels are changed in bloodstream and the intestines are required along with characterizing these changes. This is because altered SCFA production might play a possible mechanistic role in disease initiation as something similar was observed in the case of Parkinson’s disease where SCFA treatment could reproduce many features of the disease in mice. In our Tgf344-AD rats, the phyla *Bacteroidetes* was found to be decreased similar to one of the human studies. Other studies have also found *Bacteroides fragilis* to be reduced in patients with cognitive impairment and brain amyloidosis [30].

The gut microbiome may influence brain activity over time through the gut brain axis and that could be happen through the vagus nerve. Vagus nerve can sense microbial metabolites in gut and may transmit a signal to the brain which may in turn elicit a response distinct from normal state [31]. There has been previous evidence showing that gut microbiome changes may influence behavioral changes affecting learning and regulate neuronal function in mice [32]. Similarly in our Tgf344AD rat model the gut microbiome changes could in turn affect neuronal function and can have a bidirectional link. Another notable change was the reduction of *Bifidobacterium* in the Tgf344-AD rats which is a well-known probiotic genus. *Bifidobacterium* has been linked with anti-inflammatory properties [33] and decrease in intestinal permeability. A previous study has also demonstrated that *Bifidobacterium* supplementation can improve cognition in an anxious mouse model of AD [34]. This change in *Bifidobacterium* was not visible at 20 months of age where aging might have obscured the genotypic changes. In our Tgf344-AD rats, *Parasutterella* which has been previously associated with irritable bowel syndrome [35] was found to be reduced at 14 months of age. A study found that increasing Parasutterella levels were correlated with decreasing low-density lipoprotein levels [36]. *Ruminococcus* is a genus which has been linked with Alzheimer’s disease in two previous studies and in our Tgf344-AD rats, they showed a similar decrease [37]. *Prevotellaceae* has been previously associated with APOE4 positive humans and transgenic mice [38], and in our Tgf344-AD rats they were found to be decreased. *Blautia* which has been linked with Alzheimer’s from clinical studies, were also found increased in the Tgf344-AD rats at 20 months of age [1]. It has also been previously associated in Parkinson’s disease and multiple sclerosis patients. *Blautia* has been seen to have an inverse association with visceral fat [39] and is found to be involved in anti-inflammatory processes. Higher *Blautia* has been associated with disturbances in glucose metabolism, type-2 diabetes and high fat diet and it was also found to be depleted in obese children [40]. The butyrate producer, *Butyricicoccus* was also increased in the Tgf344-AD rats at 20 months of age.

Another interesting observation was that a lot of taxa changes were observed in the family *Lachnospiraceae* in the Tgf344-AD rats when compared to control rats. Similar changes have also been reported in a human Alzheimer’s microbiome study [37]. This may indicate a possible common phenomenon occurring in both patients and the preclinical rat model. *Lachnospiraceae* are major producers of SCFA’s including butyrate and form an important part of the gut microbiota. *Turicibacter* has been previously found to be increased with aging in other studies [41, 42] and it is also correlated with higher fat mass [43]. *Romboutsia* has been termed a harmful bacteria with increases observed in certain disease states [44]. Changes in the family level due to aging such as *Erysipelotrichaceae, Peptostreptococcaceae* were also found to be changed in other aging microbiome studies in mice [45, 46].

In summary, we found gut microbiota changes in the Tgf344-AD rats when compared to age-matched control WT rats with taxonomic changes observed at various levels. The aging-associated changes in the gut microbiome was prominent and shows the important role aging plays in disrupting homeostasis in the body.

## Abbreviation

AD: Alzheimer’s disease
